# Retroactive Attentional Shifts Predict Performance in a Working Memory Task: Evidence by Lateralized EEG Patterns

**DOI:** 10.3389/fnhum.2018.00428

**Published:** 2018-10-18

**Authors:** Anna Göddertz, Laura-Isabelle Klatt, Christine Mertes, Daniel Schneider

**Affiliations:** Leibniz-Research Centre for Working Environment and Human Factors, TU Dortmund, Dortmund, Germany

**Keywords:** working memory, attention, EEG, CDA, retro-cue, performance

## Abstract

Shifts of attention within working memory based on retroactive (retro-) cues were shown to facilitate performance in working memory tasks. Although posterior asymmetries in the EEG, such as the contralateral delay activity (CDA), have been used to study the active storage of lateralized working memory representations, results on the relation of such asymmetric effects to retro-cue benefits remain inconclusive. We recorded EEG in a retro-cue working memory task with lateralized items and a continuous performance response. Following either a selective or neutral retro-cue, participants adjusted the orientation of a central memory probe to the cued item. Selective retro-cues elicited an early posterior contralateral negativity (PCN), anterior directing attention negativity (ADAN) and a later modulation of CDA indicating that active storage was concentrated on the cued information. By dividing all trials into three within-condition performance quantiles, we could further show that high working memory accuracy was associated with a sustained increase of the CDA effect following the retro-cue. These results suggest that focusing resources on the active storage of relevant representations is an important factor regarding retro-cue benefits in working memory tasks.

## Introduction

Working memory comprises a set of functions that allow for the active storage of information in order to make it accessible for higher-level cognitive operations (Baddeley and Hitch, 1974; Baddeley, 1996). As the capacity of working memory is limited, goal-directed behavior requires to keep only those mental representations in an activated state that are relevant for our current action. This entails shifting the focus of attention within working memory on currently relevant contents and thus de-focusing no longer relevant information. These attentional mechanisms can for example be studied by so-called retroactive cuing (or retro-cuing) paradigms. Despite a growing body of research on the underlying cognitive mechanisms of attentional selection in the mnemonic space, there is only sparse evidence for a direct link between those mechanisms and behavioral performance. The present study made use of lateralized effects in the EEG to investigate the cognitive mechanisms underlying retroactive attentional orienting and their relation to working memory accuracy, based on within-subject comparisons of task performance quantiles.

Similar to the benefits resulting from the cuing of a subset of items prior to the presentation of to-be-remembered information, retro-cues presented after encoding were shown to lead to higher accuracy and faster responses in working memory tasks (Oberauer, 2002; Griffin and Nobre, 2003). There are, however, several explanations for such retro-cue benefits that do not need to be mutually exclusive (Souza and Oberauer, 2016). In particular, shifting the focus of attention to a working memory representation has been associated with the passive decay or with an active removal of the irrelevant information, both resulting in a release of previously occupied resources that can in turn be concentrated on the cued contents or be used for the encoding of new information (Souza et al., 2014b). This view has received support by studies revealing stronger retro-cue benefits compared to no-cue conditions when the memory array contained an increasing number of non-cued items (Sligte et al., 2008; Souza et al., 2014a; van Moorselaar et al., 2015). Additionally, working memory tasks including invalid retro-cues indicated behavioral costs when non-cued items were probed, compared to neutral cue conditions (Astle et al., 2012; Gunseli et al., 2015; van Moorselaar et al., 2015; Gressmann and Janczyk, 2016). For example, Williams et al. (2013) could show that when a to-be-forgotten item was unexpectedly probed, participants’ performance dropped to chance level. This indicates that storage was concentrated on the relevant working memory contents, while irrelevant information was dropped from memory.

However, while behavioral findings indicate that the retro-cue benefit is in parts related to a bias of working memory resources toward the storage of cued contents, EEG/MEG studies revealed differing results regarding the underlying cognitive processes. Kuo et al. (2012) used contralateral delay activity (CDA; Vogel and Machizawa, 2004) as an indicator of active storage in a retro-cue based working memory paradigm. They could show that reducing the number of focused working memory representations led to a decrease in CDA amplitude compared to a neutral cue condition, reflecting the reduction of the working memory resources invested in the active storage of information (see also: Duarte et al., 2013). Also a study by Williams and Woodman (2012) revealed a modulation of CDA following a retro-cue. A memory array containing three items at each side of fixation was followed by a retro-cue indicating only the right or left-sided items as further on relevant. While there was no lateralized effect following the memory array (CDA to the left and right hemifield canceled each other out), it was present following retro-cue presentation. More specifically, there was a more negative going deflection contralateral vs. ipsilateral to the cued hemifield. However, this modulation was revealed in a change detection paradigm without a modulation of set-size. Since the side of to-be-processed stimulus changes and retro-cue direction were confounded, it is unclear what exactly CDA was related to. It was not possible to dissociate event-related potential (ERPs) effects associated with selective storage of memory representations from the preparatory attentional bias toward the relevant side of the upcoming change display.

In a more recent study, our group presented a retro-cue followed by a central memory probe with varying stimulus onset asynchronies (300, 400, 600, 1,000 and 1,800 ms; Schneider et al., 2016). We observed correlates of attentional orienting toward the relevant working memory contents: there was a transient posterior contralateral negativity (PCN or also N2pc; Luck and Hillyard, 1994a, b) and an anterior contralateral negativity (anterior directing attention negativity or ADAN; Eimer et al., 2002) peaking at around 300 ms following the retro-cue and reflecting the orienting of attention toward the cued lateralized memory items. Furthermore, we observed a transient increase in positivity contralateral to the cued items following PCN. This effect was interpreted as an inhibitory process to guarantee a shift of attention back to central fixation prior to probe presentation (see also Sawaki et al., 2012; Mertes et al., 2016, 2017). Critically, there was no evidence of a modulation of CDA in the late part of the delay interval for longer SOA conditions (600, 1,000 and 1,800 ms). Thus, as CDA is considered as an indicator for the active storage (or online storage; Luck and Vogel, 2013; Luria et al., 2016) of visuo-spatial information in working memory, this would suggest that retro-cues led to a retroactive attentional shift without necessarily implying a bias of working memory resources toward the storage of the cued contents (although the retro-cues were 100% valid). Still, while our study, as well as a couple of others (e.g., Myers et al., 2015; Schneider et al., 2015) failed to clearly indicate a retroactive modulation of CDA, evidence for a positive correlation between the amplitude of hemispheric ERP asymmetries following a retro-cue and retro-cue benefits in behavioral performance has been provided by Duarte et al. (2013) and Heuer and Schubö (2016).

**Figure 1:**
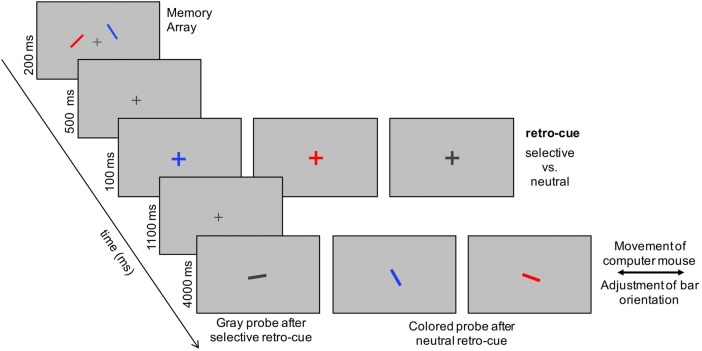
Example of the current experimental design. Participants had to memorize the orientation of two bars presented to the left and right of a central fixation cross. In two thirds of the trials, a retro-cue, in terms of an enlarged presentation of the fixation cross, indicated the color of the to-be-probed item orientation. In one third of the trials, a neutral retro cue (i.e., an enlargement of the dark gray fixation cross) was presented. In retro-cue trials, the memory probe consistent of a randomly oriented bar displayed in gray that had to be adjusted in orientation by moving the computer mouse with the right hand. The left computer mouse button had to be pressed when orientation adjustment was finished. In neutral cue trials, the probe was presented in either red or blue to indicate the to-be-reported orientation.

The current investigation tries to further disentangle the relation between retroactive cuing benefits and the above-described cognitive processes reflected in lateralized ERP effects. For this purpose, we presented a memory display with two lateralized bars in different colors (red vs. blue). This memory display was followed by a retro-cue indicating the red vs. blue bar as relevant, or by a neutral cue implying a focus on both items. Finally, a central memory probe was presented that had to be adjusted in orientation to match the cued bar (see Figure 1). Critically, following a selective retro-cue, the memory probe did not contain any additional target-related information. Thus, participants had to rely on the cue to successfully perform the task. In case of a prior neutral retro-cue, the relevant memory item was indicated by the color of the probe. With respect to the current research question, this design offers an additional important advantage: due to the continuous measure of working memory performance, it is possible to measure the accuracy of the orientation adjustment in each trial and to create accuracy quantiles within each experimental condition. This, in turn, allows for relating working memory accuracy to lateralized EEG parameters reflecting retroactive attentional orienting and selective storage of information in working memory.

First, we hypothesized that selective retro-cues (red vs. blue) should lead to the retroactive orienting of attention toward the respective mental representation. According to prior studies (Myers et al., 2015; Schneider et al., 2015, 2016; Schneider et al., 2017a), this should be reflected by the ADAN effect in the ERP. Higher efficiency in the retroactive selection processes should accordingly be associated with a stronger anterior contralateral vs. ipsilateral negativity. Second, as shown in previous research (Schneider et al., 2015, 2016; Schneider et al., 2017a), a retro-cue should be associated with a posterior contralateral positivity (PCP) following attentional selection. This effect has been associated with an inhibitory process guaranteeing the re-allocation of spatial attention to central fixation (Sawaki et al., 2012; Mertes et al., 2016). If this mechanism benefited performance, we would expect an increase of the contralateral vs. ipsilateral positivity over posterior sites with task accuracy.

In contrast to these transient ERP effects associated with attentional selection and subsequent inhibition, sustained posterior asymmetries in the ERP are of central importance regarding the question of the contribution of a retroactive modulation of working memory storage to the retro-cue benefit. Previous research indicated that just like ADAN, also early PCN should be related to selection processes following a retro-cue (Myers et al., 2015; Schneider et al., 2015, 2016; Heuer and Schubö, 2016). However, there is a problem in separating the functional meaning of this early posterior asymmetry from later sustained asymmetries when both effects are evident. As PCN and CDA have a comparable posterior topography, it is difficult to conclude when the early orienting or selection process ends and when active storage of information in working memory starts. Irrespective of this issue, we hypothesize a sustained PCN until the onset of the memory probe to qualify as a correlate of working memory storage. This storage of relevant information or the drop of irrelevant contents from active storage should directly contribute to the quality of the representation of the later-on reported item. Compared to the focusing on all items from the memory display, it would provide working memory resources that could be concentrated on the storage of the relevant item (thereby also reducing inter-item interference in working memory; Makovski et al., 2008; Souza and Oberauer, 2016) or used for item retrieval during memory probe processing (Souza et al., 2014a). Thus, the amplitude of a sustained lateralized effect in form of a CDA should be related to performance, with an ascending amplitude for increasing task accuracy. This would also explain why prior studies did not find a consistent retroactive modulation of CDA. The drop of non-cued representations is not a prerequisite for task performance in general, especially not when overall working memory load is low. However, this process should still lead to performance increases when it is required to create an exact copy of the cued item from memory. Such effects cannot be well depicted in tasks with dichotomous response categories (e.g., recognition or change detection tasks) or without focusing on high-performance trials in tasks with a continuous response scale.

## Materials and Methods

### Participants

Fourteen participants (mean age = 24.5 years; SD = 3.37; seven females) took part in the experiment. All participants were right-handed and had normal or corrected to normal vision. As reported in a screening questionnaire, none of the participants suffered from neurological or psychiatric diseases. Color blindness was ruled out by means of the Ishihara test for color blindness. Participation was rewarded by course credit or payment of 10€ per hour. The study was approved by the ethic committee of the Leibniz Research Centre for Working Environment and Human Factors and was conducted in accordance with the Declaration of Helsinki. Participants provided informed written consent before the experiment started. Parts of the data resulting from this experimental design were already published in the scope of another research question (Schneider et al., 2017a).

### Stimuli and Procedure

The experiment was run on a 22-inch CRT monitor (100 Hz) with a viewing distance of 150 cm. A ViSaGe MKII Stimulus Generator (Cambridge Research Systems, Rochester, UK) was used for stimulus presentation. At the beginning of each trial, a fixation cross (15 cd/m^2^) was presented on a gray background (luminance of 25 cd/m^2^). Then, a memory array was presented for 200 ms, including two differently colored bars (0.1 by 1° of visual angle; see Figure 1). The bars were either red or blue and presented with 25 cd/m^2^. The stimulus position on the left and right side was randomly drawn out of three positions (1.023° above or below fixation or the position on the vertical meridian). Distance to fixation was 1.5° for all six positions. The orientation of the bars was randomized, but had to differ by at least 15° in each trial. This was done to preclude that two nearly identically orientated items, though spatially separated, would be stored as only one common working memory representation. Afterwards, the retro-cues were presented as changes of the central fixation cross to either a red or blue (25 cd/m^2^ each) enlarged cross. The neutral retro-cue was displayed as an enlargement of the dark gray fixation cross. The red and blue retro-cues implied a selective retroactive shift of attention to the left or right memory item and were presented in 1/3rd of the trials each. The remaining trials (1/3rd) included the neutral retro-cue requiring a focus on both items. The retro-cues were chosen randomly, displayed for 100 ms, and followed by a 1,100 ms retention period. Finally, a memory probe was presented at the position of the central fixation cross for 4,000 ms. In selective retro-cue trials, the probe was a randomly oriented bar displayed in gray (15 cd/m^2^) that had to be adjusted in orientation to the cued memory item. In the neutral trials, the to-be-reported orientation was indicated by drawing the memory probe in either red or blue (50% of trials each; randomized). Orientation adjustment was accomplished by moving the computer mouse to the left or right. Participants were instructed to press the left mouse button with their index finger when they were satisfied with their orientation adjustment and to complete this task within 4,000 ms. We used an inter-trial interval of 500–1,000 ms subsequent to the probe display. This interval was chosen on a random basis from trial to trial. The experiment consisted of 600 trials divided into four blocks of 150 trials. A 2-min break was made between the blocks in order to prevent fatigue in the course of the experiment.

In order to train the participants in adjusting the memory probe orientation within the 4,000 ms interval, every participant took part in a training session prior to the main experiment. Two bars (1° by 0.1°) were presented lateral to a central fixation cross. One bar presented on the left or right side was either blue or red (25 cd/m^2^). It was shown at one of the memory array positions (see above). The orientation of this bar was random. The second bar on the contralateral side was rotatable by mouse movement and had to be matched in orientation to the red/blue bar. It was presented in gray with 40 cd/m^2^ and always on the vertical meridian at a distance of 1.5° to fixation. The orientation adjustment was accomplished by moving the computer mouse to left or right with the right hand. Like in the main experiment, this orientation adjustment had to be completed within 4,000 ms. The stimuli remained present during this period. For completing the training session, the final 100 trials of a block of 150 trials had to be passed with a mean angular error below 9°. Three participants required a second training block to reach this level of performance.

### Data Analyses

#### Behavioral Data

The angular error was calculated by subtracting the adjusted probe orientation from the orientation of the bar cued in the memory display. The raw angular error refers to the absolute difference and therefore does not include information about the direction of the angular error. The largest possible error was 90°. As an indicator for the overall quality of the working memory representations at retrieval, we additionally calculated the standard deviation (SD) of the raw angular error. The CircStat toolbox (Berens, 2009) for MATLAB^®^ was used for adjusting raw angular error and SD values for circular data. Only trials with a mouse click (i.e., response confirmation) were used to calculate the raw angular error and the estimated parameters. The average rate of button press trials was 98.86% (SD = 2.02%).

Behavioral parameters were analyzed using a within-subject analysis of variance (ANOVA) with the factor *retro-cue* (selective vs. neutral).

#### EEG Data

EEG was recorded by means of 60 Ag/AgCl active electrodes (ActiCap; Brain Products, Gilching, Germany). Electrodes were affixed across the scalp according to the extended 10/20 System (Pivik et al., 1993). The measurement of eye movements was accomplished by means of an electrode applied above and below the left eye (vertical EOG) and at the outer canthi of each eye respectively (horizontal EOG). A BrainAmp DC-amplifier recorded EEG and EOG with a frequency of 1,000 Hz and an online 250 Hz low-pass filter. The reference electrode during recording was at P9 and the ground electrode was set to midline electrode FPz. Impedance was kept below 10 kΩ during recording.

MATLAB^®^, EEGLAB (Delorme and Makeig, 2004) and ERPLAB (Lopez-Calderon and Luck, 2014) were used for further analyses. Data were re-referenced offline to the average signal of all electrodes (average reference). A 0.5 high-pass (6601 points; transition band width 0.5 Hz; −6 dB at 0.25 Hz) and 30 Hz low-pass FIR filter (441 points; transition band width 7.5 Hz; −6 dB at 33.75 Hz) were applied and data were then divided into segments ranging from 1,000 ms before to 3,000 ms after presentation of the initial memory array, with a 200 ms pre-stimulus baseline period. We used independent component analysis (ICA) to correct for eye blinks, vertical and lateral eye movements, and generic data discontinuities. Artifacted ICs were detected by means of ADJUST (a completely automatic algorithm; Mognon et al., 2011). Furthermore, single dipoles were estimated for each IC by means of a boundary element head model (Fuchs et al., 2002). The ICs with a dipole solution with more than 40% residual variance were also excluded, because scalp maps of artifacted ICs do usually not resemble the projection of a single dipole (Onton and Makeig, 2006). Segments with remaining artifacts were removed through automatic epoch rejection (threshold limit: 1,000 μV, probability threshold: 5 SD, Max. % of trials rejected per iteration: 5%) implemented in EEGLAB.

Only trials containing a mouse-button press were included in further analyses. This button press indicated that participants judged their orientation adjustment as adequate. As we were interested in the relation of lateralized EEG effects following the retro-cue to behavioral accuracy, we further calculated three data quantiles according to performance within experimental conditions (selective retro-cue to the left vs. right side; neutral retro-cue with left vs. right items probed) for each participant. Thus, based on the raw angular error in each trial, data were divided into good, medium and bad trials.

##### Event-Related Potentials

###### Retro-Cue

ERP analyses were limited to the selective retro-cue condition since lateralized effects were expected to occur in this condition only. We first calculated the contralateral and ipsilateral portions of the ERP irrespective of performance quantiles. This was done by averaging across trials with cued memory items on the right side and left-sided electrodes and cued memory items on the left side and right-sided electrodes (i.e., contralateral). The ipsilateral portion was accordingly calculated by averaging across trials with cued memory items on the left side and left-sided electrodes and cued memory items on the right side and right-sided electrodes. Posterior contralateral vs. ipsilateral ERPs were analyzed as the average signal from parieto-occipital, parietal and temporo-parietal lateral sites (PO7/8, P7/8, TP7/8), as prior investigations usually revealed a broad scalp distribution of posterior asymmetric ERP effects in the context of retroactive cuing working memory tasks (Schneider et al., 2015, 2016). Three components were investigated over lateral posterior sites: PCN (Luck and Hillyard, 1994a, b), CDA (Vogel and Machizawa, 2004) and PCP (Schneider et al., 2015, 2016, 2017a). ADAN (Eimer et al., 2002) was measured over lateral anterior electrodes (averaged across FC3/4 and C3/C4). We chose this combination of electrodes as prior studies indicated that while the ADAN effect usually has a lateral fronto-central maximum, it can also be observed at more posterior lateral channels (Nobre et al., 2000; Van Velzen et al., 2006).

Time windows for the asymmetric ERP effects were determined as follows: we measured the time point when the area under the contralateral minus ipsilateral difference wave reached 50% (fractional area latency or FAL_50_) in a time window from 300 ms to 600 ms following the retro-cue for PCN and ADAN, 600–900 s for PCP and between 900 and 1,200 ms for CDA. Fractional area latency might be closely related to the peak of the lateralization effect itself, resulting in high probabilities to bring up significant effects when centering the test windows around the FAL_50_ values. Thus, we specified the FAL_50_ separately for each participant using a leave-one-dataset-out technique: for participant 1, we calculated the FAL_50_ of each component (see above) based on data from participants 2–14, for participant 2, we calculated the FAL_50_ based on the data of participants 1 and 3–14, and so on. Resulting FAL_50_ values are summarized in Table 1. Lateralization effects for each participant were then measured as the area under the negative (PCN, ADAN, CDA) or positive part (PCP) of the contralateral minus ipsilateral difference wave in a 200 ms time window centered on these individual FAL_50_ values. Thus, for every subject individual time windows were used for analyses, based on each but the respective subject of the sample.

**Table 1:** Summary of resulting individual fractional area latencies (ms) locked on the onset of the selective retro-cue.

	Fractional area latencies			
	Min	Max	Mean	SD
ADAN	421	434	427	3.58
PCN	443	452	446	3.21
PCP	701	716	705	4.04
CDA	1058	1120	1173	15.23

A special procedure was furthermore required for evaluating if the measured areas actually differed from chance level (Sawaki et al., 2012). This was the case, because the area measures were only based on either positive or negative deflections. Thus, noise in the data always biased the areas toward values greater than zero. To this extent, we randomly assigned the conditions with a cued item on the left vs. right side to the trials and measured the negative (PCN, ADAN, CDA) and positive areas (PCP) based on this random pattern. This procedure was run 1,000 times and resulted in a random distribution of area values for each ERP component based on the varying assignments of left and right cue positions. The area measures were based on the 200 ms time windows centered on the individually calculated FAL_50_ for each subject. If the area value measured in the original data was higher than 95% of this distribution, it was considered to differ significantly from chance.

The relation of retroactive attentional orienting to response error in the working memory task was approached by comparing PCN, PCP, ADAN and CDA effects between the three performance quantiles measured within experimental conditions (i.e., retro-cue to the left vs. right side). ERP effects were estimated for the time windows and electrode clusters described above. We intended to prove a linear relationship between the ERP effects and working memory accuracy. Therefore, we applied linear mixed-effects models with *performance* (good, medium, bad) as a fixed effect under the assumption of different random intercepts for each participant. This led to the following formula for the model fits (1 refers to the intercept and ε to the residual error in the model):

Effect (ERP) ~ 1 + performance + (1|participant)+ε

To test for significant linear courses of the ERP effects (PCN, ADAN, PCP and CDA) across performance quantiles (i.e., the fixed effect), we used an ANOVA for linear mixed-effects models and the Satterthwaite method (Satterthwaite, 1941) to estimate the respective degrees of freedom. In order to correct for multiple analyses, we further provide the corrected *p*-values based on the false discovery rate (FDR) method (Benjamini and Hochberg, 1995). The variance in intercepts in our models implies that participants differ regarding the baseline of the effect of interest. In order to test if this assumption was applicable, we provide the 95% confidence intervals around the expected value. The variance in intercepts across participants was considered significant when the confidence intervals did not include zero.

###### Memory Array

In addition, the ERP effects following the memory array were considered in order to exclude the possibility that a relation between task accuracy and ERP asymmetries following the retro-cues was related to a general variability in cognitive resources applied to the task. For this purpose, the selective and neutral retro-cue conditions were combined and a variety of ERP components following the initial memory array were selected for further analyses: that is, the posterior P1 and N1 as markers for the sensory processing of the memory array, the fronto-central N2 as a correlate of cognitive control during memory array processing (van Veen and Carter, 2002; Folstein and Van Petten, 2008), the parietal P3 (P3b) reflecting the encoding of information into working memory (Donchin and Coles, 1988; Polich, 2007) and the posterior negative slow wave (NSW) as a marker for working memory resources invested in the storage of the two items (Ruchkin et al., 1992). Time windows and electrodes for measuring mean amplitudes were selected based on the ERPs collapsed across performance quantiles. P1 and N1 were studied at electrodes PO7 and PO8 where the maximum positivity or negativity was observed. Mean amplitude time windows (20 ms) for statistical analyses were centered at the positive (P1) and negative (N1) peaks in the grand average. P1 mean amplitudes were accordingly measured from 118 ms to 138 ms and N1 mean amplitudes from 182 ms to 202 ms. Mean amplitudes for fronto-central N2 were measured at electrode FCz and within a 40 ms interval centered at the grand average N2 peak at 319 ms. In a comparable way, P3b was recorded at Pz where its effect was maximal and with a mean amplitude time window from 328 ms to 368 ms. Posterior NSW was measured at PO1 where the maximum negativity was observed in the interval following P3b and before the onset of the retro-cues. Due to the sustained nature of this slow wave, a 200 ms time window centered at the negative peak in the interval after P3b was used for measuring mean amplitudes (602–802 ms). For all ERP components, mean amplitudes were measured within each of the three within-subject performance quantiles. The quantiles were computed separately for each experimental condition (selective retro-cue to the left vs. right side; neutral retro-cue with left vs. right items probed). Afterwards, linear mixed effects models were fitted on the data with the above-mentioned components as dependent variables, *performance* (good, medium, bad) as a fixed effect, and a random intercept for participants.

Furthermore, we wanted to assess whether the relation between posterior asymmetries in the ERPs following the retro-cues and performance differed from the relation between the ERPs following the memory array and performance. For this purpose, each ERP effect was z-transformed across datasets and linear mixed effects models with *performance* (good, medium, bad), *component* (memory array ERPs vs. posterior asymmetry) and the interaction of these factors as fixed effects were applied under the assumptions of a random intercept for participants. Separate models were fitted for each memory array ERP effect. The posterior asymmetry following the retro-cues was measured as the mean amplitude of the contralateral minus ipsilateral difference wave at the posterior lateral channel cluster (PO7/8, P7/8, TP7/8) in an interval from 600 ms to 1,200 ms after retro-cue onset. Adjusted *p*-values for multiple analyses were calculated by means of FDR correction (Benjamini and Hochberg, 1995).

All statistical analyses were done using the Statistics and Machine Learning Toolbox for Matlab^®^. The datasets generated during and/or analyzed during the current study are available from the corresponding author on reasonable request.

## Results

### Behavioral Data

**Figure 2:**
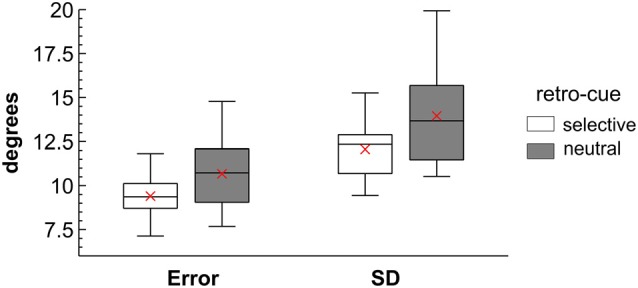
Behavioral results presented as boxplot diagrams. Shows the mean absolute angular error and the standard deviation (SD) of the error for the selective and neutral retro-cue conditions. The mean is represented by the red cross. The boxplots indicate the median, the 25% and 75% percentiles and the minimum and maximum values of the distributions.

Behavioral analyses focused on the comparison of performance between the selective retro-cue condition and the neutral condition without a selective shift of attention prior to presentation of the memory probe. The raw angular error was larger in neutral (*M* = 10.63°, *SD* = 1.16) compared to selective retro-cue trials (*M* = 9.38°, *SD* = 1.17), *F*_(1,13)_ = 19.69, *p* < 0.001, $\eta_{\text{p}}^{2}$ = 0.60. In addition, the standard deviation of the error distribution was larger in the neutral (*M* = 13.90°, *SD* = 1.84) than in the selective retro-cue condition (*M* = 12.05°, *SD* = 1.77), *F*_(1,13)_ = 12.82, *p* = 0.003, $\eta_{\text{p}}^{2}$ = 0.50 (see Figure 2).

### EEG Data

#### Event-Related Potentials

##### Retro-cue

**Figure 3:**
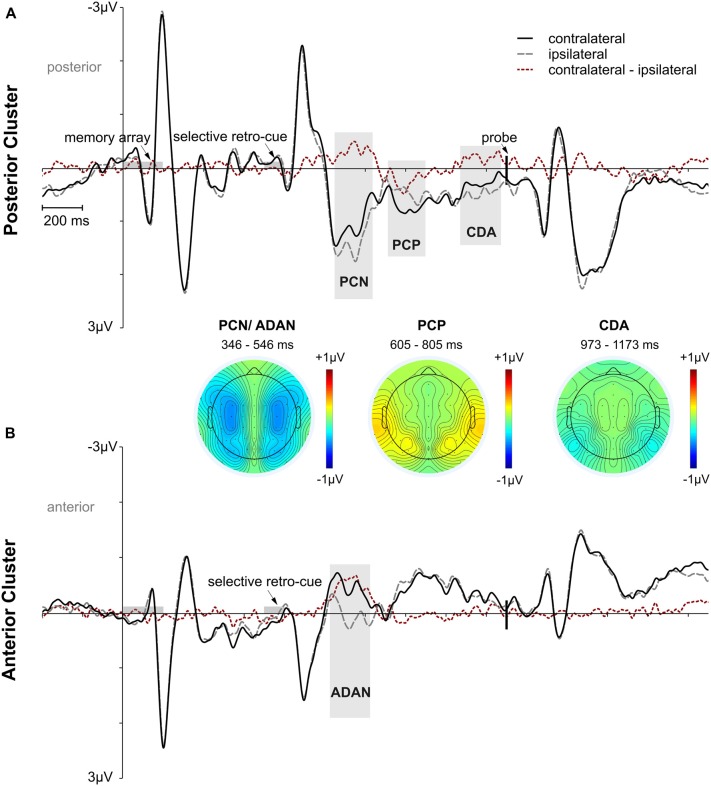
Overall event-related potential (ERP) effects time-locked to memory array onset for the posterior and anterior electrode clusters. Time intervals for memory array and retro-cue presentation are highlighted in gray. The vertical line marks the onset of the probe stimulus. Panel **(A)** shows posterior contralateral negativity (PCN), posterior contralateral positivity (PCP), and contralateral delay activity (CDA) effects for the selective retro-cue condition. Panel **(B)** depicts anterior directing attention negativity (ADAN) for the selective retro-cue condition. Additional topographies are depicted for the 200 ms analysis time windows centered on the mean FAL_50_ across participants. The PCN and ADAN effects are shown together in one topography. The red dotted lines reflect the contralateral minus ipsilateral difference waves.

**Figure 4:**
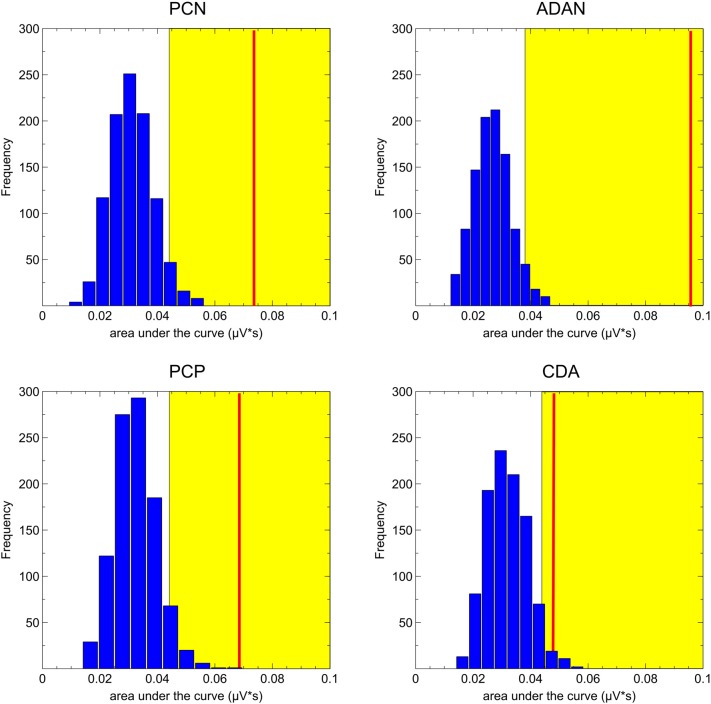
Results of the randomization tests for the ERP asymmetries. The blue bars depict the random distribution of the area under the curve values. The yellow area specifies all values higher than 95% of the random distribution and the red line indicates the observed area under the curve value averaged across participants. The asymmetries were considered as statistically significant when the red line lay within the yellow area.

ERP analyses were restricted to the selective retro-cue condition, as we were interested in how retroactive attention during the storage of information in working memory modulates later performance. Regarding the lateralized ERP effects following the retro-cue, we first tested whether asymmetric effects in the posterior (PO7/8, P7/8, TP7/8) and anterior (FC3/4, C3/4) contralateral vs. ipsilateral ERPs were evident in the data (see Figure 3). The lateralized effects in the ERP were assessed by means of area under the negative (PCN, ADAN, CDA) or positive part or PCP of the contralateral minus ipsilateral difference waves within 200 ms intervals adjusted for each subject and component. The randomization tests illustrated in Figure 4 were used for testing if a lateralized effect was evident (see “Materials and Methods” section for more details). Statistically reliable PCN and ADAN effects were shown. Furthermore, we reliably observed a PCP following PCN and ADAN. Also, a CDA effect was evident.

**Figure 5:**
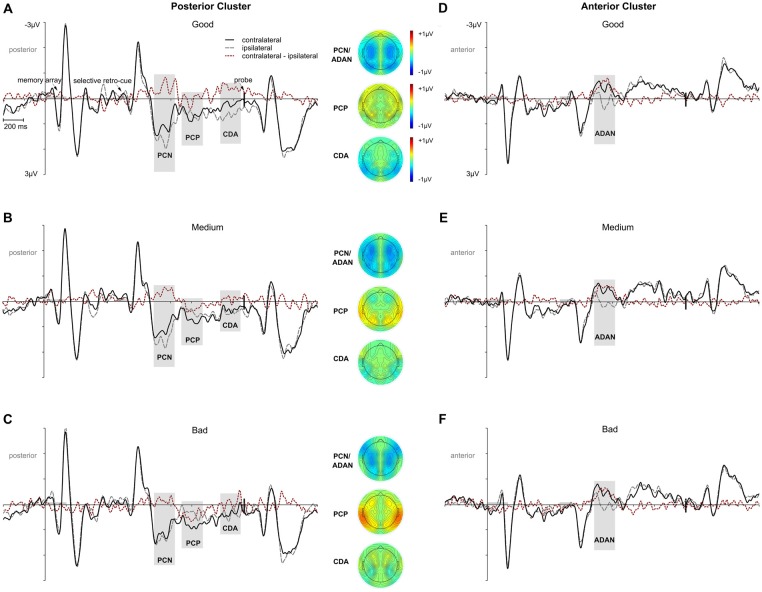
ERP effects as a function of performance for the selective retro-cue condition. Presentation times of the memory array and the retro-cue are highlighted in gray. The onset of the probe array is indexed by the vertical black line. Panels **(A–C)** depict PCN, PCP and CDA effects for the posterior cluster separately for trials with good, medium, or bad performance. The ADAN effect at anterior sites as a function of performance is shown in **(D–F)**. Separate topographies are given for the 200 ms intervals centered on the mean of the resulting individual participants’ FAL_50_. The red dotted lines reflect the contralateral minus ipsilateral difference waves.

Further analyses were conducted for investigating to what extent these ERP effects in the context of retroactive attentional orienting differed as a function of the response error. Thus, we calculated three performance quantiles for trials with retro-cues toward left and right items, respectively, and fitted linear mixed-effects models for each ERP effect with *performance* as a fixed effect and a random intercept for participants (see “Materials and Methods” section for more details). Every model revealed a significant random intercept for participants (i.e., 95% confidence intervals did not include zero). The *performance* fixed effect was not reliable for PCN, *F*_(1,28)_ = 1.633, *p* = 0.212, *p*_adj_ = 0.283 (95% CI: −0.037, 0.008). Similarly, for ADAN, there was no statistically reliable *performance* effect, *F*_(1,28)_ = 0.556, *p* = 0.462, *p*_adj_ = 0.462 (95% CI: −0.028, 0.013; see Figures 5D–F). However, there was a linear relation between performance quantiles and PCP amplitudes, *F*_(1,28)_ = 8.346, *p* = 0.007, *p*_adj_ = 0.030 (95% CI: 0.006, 0.034). The positive area under the difference curve decreased with increasing task accuracy (i.e., a negative shift in the contralateral minus ipsilateral difference; see Figures 5A–C). The same effect was observed for CDA, with a *performance* effect due to higher negative area under the curve values with increasing task accuracy, *F*_(1,28)_ = 6.340, *p* = 0.018, *p*_adj_ = 0.036 (95% CI: −0.036, −0.004; see Figures 5A–C).

Overall, the data pattern across all components suggested that performance differences in ERP asymmetries were related to a posterior contralateral negative shift starting around the PCP time window and lasting until memory probe presentation. We further approached this effect by testing a linear mixed-effects model on the same data, but now with the mean amplitude of the contralateral minus ipsilateral difference at posterior sites (PO7/8, P7/8, TP7/8) within an interval from 600 ms to 1,200 ms after retro-cue onset (comprising the time windows of PCP and CDA) as dependent variable, the performance quantiles as a fixed effect, and a random intercept for participants. This model provided a significant random intercept for participants (95% CI: 0.003, 2.297) and a statistically reliable effect of *performance*, *F*_(1,28)_ = 7.505, *p* = 0.01, *p*_adj_ = 0.05 (95% CI: 0.051, 0.339), confirming our assumption of a linear course underlying the ascending negative shift in the contralateral minus ipsilateral difference wave with increasing task accuracy (bad < medium < good; see Figures 5A–C).

As an additional proof for the relation between task accuracy and ERP patterns, the reliable appearance of the anterior and posterior asymmetries was again tested by means of the randomization approach in each performance quantile. We provide the mean area values for each component and the critical value (95% of the distribution) in Table 2. The ADAN and PCN effects were statistically significant for all performance quantiles. While a PCP effect was only observed in medium and bad trials, later CDA was exclusively evident in the good performance quantile.

**Table 2:** Critical and observed values of the area permutation analyses for anterior directing attention negativity (ADAN), posterior contralateral negativity (PCN), posterior contralateral positivity (PCP) and contralateral delay activity (CDA).

	Performance					
	Good		Medium		Bad	
Component	Critical	Observed	Critical	Observed	Critical	Observed
ADAN	0.069	0.110*	0.064	0.107*	0.068	0.095*
PCN	0.079	0.107*	0.075	0.083*	0.079	0.079*
PCP	0.081	0.078	0.075	0.104*	0.081	0.101*
CDA	0.081	0.101*	0.076	0.044	0.080	0.061

*Values are displayed for the performance quantiles. Significance of the effects is assumed when the observed value exceeds the critical value (indicated by an asterisk)*.

##### Memory Array

**Figure 6:**
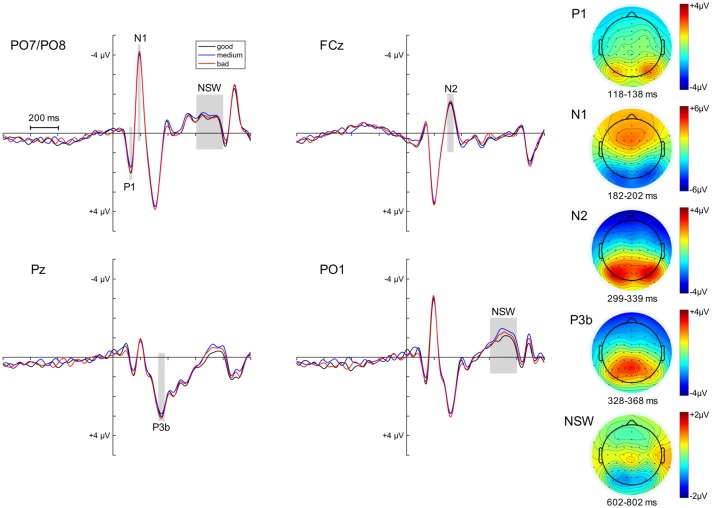
ERP effects following the memory array. The ERPs subsequent to the memory array and before cue presentation are depicted separately for high, medium and low performance quantiles at PO7/PO8 (combined), FCz, Pz and PO1. The analysis time windows for the components of interest (posterior P1/N1, fronto-central N2, parietal P3 (P3b) and posterior negative slow wave or NSW) are highlighted by the light gray squares. The topographies for each component (averaged across performance quantiles) are displayed on the right side of the figure.

We also examined to what extent variability in the ERPs following the memory array was related to task accuracy. This was again done by means of three quantiles based on the raw angular error within each experimental condition (retro-cue left vs. right; neutral cue with probe left vs. right). The ERP effects of posterior P1, N1, fronto-central N2, P3b and posterior NSW following the initial memory array and the respective topographies can be observed in Figure 6.

Linear mixed-effects models were fitted on the data with the above-listed ERPs as dependent variables, the performance quantiles as a fixed effect, and a random intercept for participants. The random intercept for participants was significant in all tested models (95% confidence intervals did not include zero). No reliable effect of *performance* was observed for any of the memory array ERPs (*p*-values > 0.17, 95% CI included zero).

A further fixed factor for *component* (memory array ERPs vs. posterior asymmetry) was introduced to the linear mixed effects models mentioned above. These analyses revealed significant *component* by *performance* interactions for N2, *F*_(1,70)_ = 5.761, *p* = 0.019, *p*_adj_ = 0.048 (95% CI: −0.901, −0.084), and posterior NSW, *F*_(1,70)_ = 7.03, *p* = 0.01, *p*_adj_ = 0.048 (95% CI: −0.887, −0.126). The *component* by *performance* interactions in the models with P1 amplitudes, *F*_(1,70)_ = 4.048, *p* = 0.048, *p*_adj_ = 0.069 (95% CI: −0.908, −0.004), N1 amplitudes, *F*_(1,70)_ = 3.782, *p* = 0.055, *p*_adj_ = 0.069 (95% CI: −0.887, 0.01), and P3b amplitudes, *F*_(1,70)_ = 3.35, *p* = 0.071, *p*_adj_ = 0.071 (95% CI: −0.848, 0.035), as dependent variables were marginally significant.

These results render it very unlikely that the observed link between performance and ERP indices of retroactive attentional orienting (see Figures 5A–C) was related to a general variability in cognitive resources applied to the task.

## Discussion

The current study investigated the relationship between ERP parameters of retroactive attentional focusing and accuracy in a working memory task based on within-subject comparisons of task performance quantiles. Therefore, we used a continuous performance working memory paradigm with endogenous retro-cues indicating one (selective retro-cue condition) or two out of two lateralized items (neutral condition) as furthermore relevant for the task. Results indicated that high accuracy in the working memory task was not related to the efficiency of retroactive selection mechanisms (i.e., no significant modulation of PCN or ADAN with accuracy), but to the selective storage of relevant visuo-spatial representations following the retro-cue, as indicated by a late modulation of the posterior CDA effect in the ERP.

On the behavioral level, the raw angular error and its standard deviation within conditions (see Figure 2) indicated an increment in working memory accuracy for the selective compared to the neutral retro-cue condition. While these effects were rather small on descriptive level, they were highly statistically significant. The rather small benefit on working memory accuracy might be related to the fact that the retro-cue reduced the number of relevant items by only 1. There are several possible mechanisms that might underlie this retro-cue benefit: for example, it might be related to the head-start of selection after the retro-cue that should facilitate the transfer of the cued representation into a response-oriented representation already prior to memory probe presentation. While our analyses did not directly focus on response plans represented in working memory, earlier studies pointed toward the relevance of such a mechanism (Myers et al., 2017; Schneider et al., 2017b). Furthermore, the selection of the cued representations might involve the release of the non-cued information from active storage in working memory. This should free up resources that can in turn be applied to the storage of the relevant items or during memory probe processing (Souza et al., 2014b). The lateralized ERP patterns addressed below provide evidence for such a mechanism.

Based on the lateralized setup of the memory array, we analyzed the contralateral vs. ipsilateral ERP waveforms following the retro-cue presentation to investigate how electrophysiological correlates of selective attention within working memory are related to the behavioral retro-cue benefit. Comparable to prior studies based on the retroactive search of target stimuli within mnemonic representations (e.g., Kuo et al., 2009), there was an early contralateral negativity over posterior lateral sites (referred to as PCN) and an ADAN effect over lateral anterior sites following the selective retro-cue. ADAN was observed across modalities in studies with spatial cues preceding the relevant information (e.g., visual, auditory, or haptic) and was thus associated with the supramodal control over the orienting of spatial attention (Eimer et al., 2002). Comparable to earlier studies, ADAN appeared as a transient asymmetry (i.e., it did clearly not last throughout the delay interval) emerging over lateral frontal sensors at about 300–500 ms after cue presentation. Thus, in line with earlier investigations on ADAN in the context of retro-cuing (Myers et al., 2015; Schneider et al., 2015, 2016), it can be interpreted as a correlate of attentional orienting in mnemonic space. However, the analysis of ADAN and PCN amplitudes did not reveal a reliable relation to task accuracy. There are several possible reasons for the lack of such a relation. First, only two items were presented in the memory array and the retro-cues were required for successful task performance (since no further cue indicating the target item was provided by the later probe). This might have led to a low variance in the orienting of attention following the retro-cue (e.g., few occurrences of erroneous orienting toward the non-cued representation or not considering the cue at all), thus reducing the possibility to predict variance in task accuracy. If this assumption holds true, a relation between ADAN (and/or PCN) amplitude and task accuracy should be evident when overall working memory load and thereby the variability in target selection is increased. This was the case in the study of Heuer and Schubö (2016). They observed a positive correlation between PCN amplitude following a retro-cue selecting one out of four items and response accuracy. Alternatively, a less plausible but still possible interpretation of results would be that the retro-cue benefit is generally unrelated to the selection mechanism reflected in ADAN. In this case, also a higher set size in the memory array would not lead to a relation between ADAN amplitude and task accuracy.

While further research will be required to clarify this relation (or rather the lack of a relation) between the efficiency of retroactive attentional selection and working memory accuracy, the current results allow for drawing a connection between the variability in working memory storage reflected by posterior ERP asymmetries and the retro-cue benefit. Note, that shifting the focus of attention and active storage in working memory are two separable processes. Selection of a representation on the one hand does not necessarily imply a high quality of the stored information. Storage, on the other hand, is possible outside of the focus of attention (e.g., Zokaei et al., 2014). The active storage of visuo-spatial information in working memory is usually approached by looking at the CDA effect in the ERP (Vogel and Machizawa, 2004; Ikkai et al., 2010; Luria et al., 2016; Feldmann-Wüstefeld et al., 2018).

In the current study, PCN was followed by a sustained contralateral negativity lasting until memory probe onset that appeared only in high performance trials. We argue that this effect corresponds to a difference in CDA amplitudes between performance quantiles. However, PCN and CDA time windows were analyzed separately, as we also observed a posterior contralateral positivity (here labeled PCP) that interrupted the contralateral negativity period. The functional relevance of this positive lateralized ERP effect is less clear. It can be assumed that it was related to an inhibitory process required to compensate for the directing of attention toward the cued side in perceptual space, necessary for the processing of the upcoming probe stimulus presented centrally (Schneider et al., 2015, 2016). Critically, if this mechanism itself would facilitate accuracy in the working memory task, we would expect an increase of the PCP effect with ascending performance. However, we observed a decrease of the PCP effect while the subsequent CDA effect was larger for higher performance quantiles. This suggests a sustained linear enhancement of posterior contralateral vs. ipsilateral activity with increasing working memory accuracy starting around the onset of the overlapping PCP effect and lasting until probe presentation (see Figures 5A–C). This is in line with earlier results showing positive correlations between the amplitude of CDA and performance benefits in retro-cuing paradigms (Duarte et al., 2013; Heuer and Schubö, 2016). While those studies used between-subjects correlations, the current design benefits from ruling out interindividual variability due to a within-subject design. Unfortunately, we cannot say with absolute certainty that the described contralateral NSW is a CDA, as we did not modulate set size. One might, for example, interpret the late contralateral negativity as a second PCN. However, if this assumption would hold true, the late contralateral negativity would reflect a shift of spatial attention in preparation for the probe display. This is very unlikely, as the probe was presented at fixation and color and location information were no longer required for task performance after processing of the 100% valid retro-cues.

Furthermore, our results raise the question whether the relation between task accuracy and the late CDA variability was specific to the use of the retro-cue for working memory updating, or whether it was instead related to a general variability in cognitive resources applied to the task (Adam et al., 2018). For example, high CDA amplitudes following the selective retro-cues might actually be related to more resources applied to the encoding of the memory array contents and not to a variability in the retroactive attentional mechanisms. As the current design did not manipulate the general demands on cognitive resources across trials, it is not possible to completely rule out this concern. However, the relation between the CDA effect and task accuracy differed from the relation between task accuracy and ERP components reflecting sensory and later cognitive processing stages referred to the memory array. It would be very unlikely to observe this pattern of results if differences in the late CDA effects across performance quantiles were based on a general variance of applied cognitive resources over the course of the experiment.

Yet, what does the relation between CDA variability and task accuracy tell us about the nature of the retro-cue benefit in working memory tasks? We argue that this question can be solved by more closely taking the functional significance of CDA into account. When pre-cues indicate the upcoming target side, for example, in change detection paradigms, CDA lasts throughout the retention interval and its amplitude varies with the number of items activated in memory. Eventually, the CDA effect reaches an asymptote when individual working memory capacity is exceeded (Vogel and Machizawa, 2004; Vogel et al., 2005; Ikkai et al., 2010). Interestingly, CDA cannot exclusively be observed in memory tasks that include delay intervals without stimulus presentation, but also when the relevant information remains within view (Tsubomi et al., 2013). This highlights that CDA is not simply linked to any kind of short-term storage. It is rather exclusively associated with keeping a mental representation in an activated or online state (Luria et al., 2016). This is exactly one of the mechanisms that is usually also ascribed to retro-cues: in a first place, cued mental representations are selected and can thus be transferred to a higher-level representational state, for guiding the later response to the probe stimulus (Myers et al., 2017; Schneider et al., 2017b). This process is essential for performing the task at hand when no further cue indicating the to-be-reported information is provided and should be reflected in the ADAN effect of the ERP. Additionally, a retro-cue might induce a change of the visuo-spatial representations activated in working memory, including the drop of irrelevant information from an activated visuo-spatial state^1^. This process was reflected by the CDA modulation in the current study. Such a mechanism should be optional for task completion. However, a lower number of activated representations should be related to lower inter-item interference and thereby facilitate retrieval processes (Vogel and Machizawa, 2004; Duarte et al., 2013). Additionally, focusing resources on the active storage of only one item was associated with the protection of this representation from sensory interference (for example by the memory probe; Makovski and Jiang, 2007). By showing that a CDA effect following a retro-cue emerged only for high-performance trials, we thus strongly support the notion that the underlying mechanism, albeit optional, still facilitates the selective drawing on the cued information during report (i.e., the adjustment of memory probe orientation) and thereby benefits task accuracy. To further strengthen this notion, future research should aim at replicating the current CDA-performance relation in a retroactive cuing task and also make use of variable delay intervals and memory array set sizes. Besides the late onset and sustained nature of the effect in the current investigation, an extension of CDA with longer delay intervals and an increase of amplitude with the number of cued items would further support the link between the observed effects and the variability in working memory storage.

In summary, we showed that endogenous retro-cues facilitated performance in a working memory task compared to a neutral condition. We furthermore showed that the benefit on task accuracy in the selective retro-cue condition was associated with an increased CDA effect in the delay interval prior to probe presentation. This association with task accuracy was neither evident in other correlates of retroactive attentional orienting (e.g., ADAN) nor in the ERPs reflecting the processing of the memory array contents. We thus conclude that focusing resources on the active storage of relevant mental representations might be an optional but still helpful process when it is required to reproduce copies of stimuli from working memory as accurate as possible.

## Author Contributions

AG and DS set up the experimental design and analyzed the behavioral and EEG data. All authors contributed to writing and reviewing the manuscript.

## Conflict of Interest Statement

The authors declare that the research was conducted in the absence of any commercial or financial relationships that could be construed as a potential conflict of interest.

## Footnotes

^1^The removal process includes the drop of color and location information of both the relevant and the irrelevant item. Note however, that it has been shown that maintenance of additional color or location information of a complex object does not modulate CDA (Woodman and Vogel, 2008; Ikkai et al., 2010; Luria and Vogel, 2011; Kursawe and Zimmer, 2015).
